# Microstrip sensor and methodology for the determination of complex anisotropic permittivity using perturbation techniques

**DOI:** 10.1038/s41598-022-06259-8

**Published:** 2022-02-09

**Authors:** Hector-Noel Morales-Lovera, Jose-Luis Olvera-Cervantes, Aldo-Eleazar Perez-Ramos, Alonso Corona-Chavez, Carlos E. Saavedra

**Affiliations:** 1 Instituto Nacional de Astrofísica, Óptica y Electrónica, Puebla, Mexico; 2 CONACYT–CICESE, Unidad Monterrey, Apodaca, Mexico; 3 Queen’s University, Kingston, Canada

**Keywords:** Electrical and electronic engineering, Sensors and biosensors, Characterization and analytical techniques

## Abstract

In this work, a sensor in microstrip technology and a methodology for measuring the real part and the imaginary part of the complex uniaxial permittivity of solid anisotropic samples are presented. The sensor is based on a pair of parallel lines coupled resonators and a cleft arranged in the coupling region which allows to hold the samples under test (SUTs). The proposed methodology relates the change in the even/odd resonance frequency with the real part of the permittivity in the vertical/horizontal direction, and the change in the Q factor of the even/odd mode with the imaginary part of the permittivity in the vertical/horizontal direction. The methodology was successfully verified with the characterization, at 2.43 GHz of anisotropic samples of printed PLA, Diclad 880, and RO4350B using the knowns materials: RT5870, PTFE and RO4003.

## Introduction

Microwave sensors are an important option for measuring the physical–chemical properties of materials, which has led to their application in different fields including biomedical^1,2^ fault detection^3^, humidity and corrosion^4,5^, position^6^, level^7^, characterization of materials^8–12^ and many others due to their low cost and high sensitivity.

The complex relative permittivity ${\stackrel{\sim }{\varepsilon }}_{r}$ is the parameter measured in many microwave sensors, since it describes the dielectric characteristics of a material which is a function of frequency

1 $${\stackrel{\sim }{\varepsilon }}_{r}\left(\omega \right)={{\varepsilon }^{{\prime}}}_{r}-j{\varepsilon }^{{{\prime\prime}}}$$

where the real part ${{\varepsilon }^{^{\prime}}}_{r}$ called the dielectric constant is a measure of the ability of a material to store electrical energy, while the imaginary part ${\varepsilon }^{{{\prime\prime}}}$ is known as the loss factor and is related to the power loss in the material. Complex permittivity can be dependent on the direction of propagation of the electromagnetic field, so that the material is anisotropic and ${\stackrel{\sim }{\varepsilon }}_{r}$ a second rank tensor shown in Eq. (2), where the components of said matrix describe the permittivity of the anisotropic medium in the different directions of space.

2 $${\stackrel{\sim }{\varepsilon }}_{r}\left(\omega \right)=\left[\begin{array}{ccc}{\stackrel{\sim }{\varepsilon }}_{xx}& {\stackrel{\sim }{\varepsilon }}_{xy}& {\stackrel{\sim }{\varepsilon }}_{xz}\\ {\stackrel{\sim }{\varepsilon }}_{yx}& {\stackrel{\sim }{\varepsilon }}_{yy}& {\stackrel{\sim }{\varepsilon }}_{yz}\\ {\stackrel{\sim }{\varepsilon }}_{zx}& {\stackrel{\sim }{\varepsilon }}_{zy}& {\stackrel{\sim }{\varepsilon }}_{zz}\end{array}\right]$$

Anisotropy in matter can present itself in different ways, some more complex than others. The simplest anisotropy is that of materials with two different permittivity values: known as uniaxial anisotropy. This kind of materials have a permittivity in the horizontal direction ${{\stackrel{\sim }{\varepsilon }}_{\parallel }=\stackrel{\sim }{\varepsilon }}_{xx}={\stackrel{\sim }{\varepsilon }}_{yy}$ different from a permittivity in the vertical direction ${\stackrel{\sim }{\varepsilon }}_{\perp }={\stackrel{\sim }{\varepsilon }}_{zz}$. Uniaxial anisotropy is of great interest to microwave engineers since many components are designed on PCB substrates. Which present this type of anisotropy, so the reliability of their designs depends largely on knowing the anisotropy of the dielectric substrates.

Several works have been reported in the literature for the anisotropic characterization of SUTs. Characterization methods can be classified as resonant methods and non-resonant methods. The resonant methods allow to obtain the characterization of SUTs in discrete frequency points while the non-resonant methods allow to know the dielectric properties of the material in a wide range of frequencies.

Non-resonant methods generally use impedance and/or complex propagation constant in transmission lines or waveguides. Some examples of non-resonant methods are those reported in previous works^11,13,14^. CPW lines are used to determine the complex uniaxial permittivity^11^ (a permittivity in the horizontal direction ${{\stackrel{\sim }{\varepsilon }}_{\parallel }=\stackrel{\sim }{\varepsilon }}_{xx}={\stackrel{\sim }{\varepsilon }}_{yy}$ different from a permittivity in the vertical direction ${\stackrel{\sim }{\varepsilon }}_{\perp }={\stackrel{\sim }{\varepsilon }}_{zz}$) of the evaluated material; the method starts from taking multiple measurements and a necessary calibration process to find the sensor's propagation constant with the SUT. In Fritsch et al.^13^, the anisotropy of a SUT is determined by phase constant measurements and rigorous analysis of the scattering characteristics of microstrip lines; it is worth mentioning that microstrip technology has the advantage that it is low cost to manufacture, integrable, can be mass manufactured, and operates from low frequency to the millimeter wave range. In Felicio et al.^14^, a rectangular waveguide characterization technique is reported which allows the permittivity to be evaluate within a narrow band with the SUTs oriented towards the three main axes within the waveguide. Where were reported both; the dielectric constant and the loss tangent ($tan\delta = {\varepsilon }^{{{\prime\prime}}}/{\varepsilon }_{r}^{^{\prime}})$ of the polylactic acid (PLA) material characterized at 40 GHz.

Among the resonant methods are those based on coupled microstrip resonators^9,15^ and methods based on cavity resonators^12,16^. In the work presented by Rautio et al.^15^ the fabrication of coupled microstrip resonators on an unknown SUT is carried out, while in the work of Morales et al.^9^ two coupled resonators are manufactured on a known substrate, and this is used as a sensor for the measurement of different SUTs. Both works were successfully tested for the determination of planar anisotropic materials at frequencies from 800 MHz to 2.45 GHz. It is important to mention that both the work of Morales et al.^9^ and that of Rautio et al.^15^ allow the determination only of the real part of the relative permittivity. In addition, the extraction methodology includes an iterative process (space-mapping) that correlates measurements with simulations of the experiment as realistic as possible, the latter demands tuning processes, computational resources, and processing time.

Alternatively, Dankov^12^ and Chen et al.^16^ proposed methods for uniaxial anisotropic characterization based on the cavity perturbation theory using cylindrical cavity resonators. In Dankov^12^, ${\stackrel{\sim }{\varepsilon }}_{\parallel }$ is obtained by means of a resonator that sustained the ${TE}_{011}$ mode, while ${\stackrel{\sim }{\varepsilon }}_{\perp }$ is obtained with another resonator that sustained the ${TM}_{010}$ mode. On the other hand, in Chen et al.^16^ a cylindrical cavity resonator with the mode ${TE}_{112}$ that has orthogonal electric field lines, allows the measurement of the complex uniaxial permittivity of SUTs. However, it is required to intervene in the cylindrical cavity by means of metallic needles to eliminate the field lines parallel to these, so that the SUT is polarized with the field lines perpendicular to the needles. It is important to mention that the works proposed by Dankov^12^ and Chen et al.^16^ require four measurements of S parameters, two being for the measurement with a standard sample and two for the SUT, intervening in the cavity as in Chen et al.^16^ or moving the sample to different cavities as in Dankov^12^. Furthermore, they have limitations at low frequencies due to the dimensions of the resonant cavity. However, they have the advantage of being able to determine the real part as the imaginary part of the permittivity using only experimental results. Additionally, these methods offer high precision due to the strong concentration of electromagnetic fields within the cavity.

In this work, a sensor, and a methodology for measuring complex uniaxial permittivity in solid samples is presented. The sensor is based on a pair of parallel line coupled resonators and a cleft arranged in the coupling region to hold the SUTs. The proposed sensor makes it possible to determine the anisotropic complex permittivity through the modes of the even and odd propagation modes. Using the even mode, it is possible to determine the permittivity of a SUT in the vertical direction, while using the odd mode it is possible to determine the permittivity in the horizontal direction of the SUT. The sensor and the proposed methodology allow the characterization of the real and imaginary part of the uniaxial permittivity, it does not require space mapping, which avoids the use of simulations in the permittivity extraction process and only needs a single measurement for the SUT and a measurement with a known standard sample. The proposed methodology was successfully verified with the characterization, at 2.43 GHz of anisotropic samples of printed PLA, Diclad 880, and RO4350B using the known isotropic material; PTFE and the knowns anisotropic materials; RT5870 and RO4003.

## Results

### Proposed sensor

**Figure 1:**
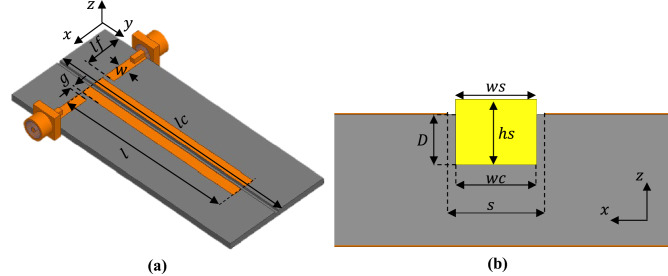
(**a**) Surface view of the proposed sensor. (**b**) Cross-sectional view of the sensor loaded with a solid SUT.

**Figure 2:**
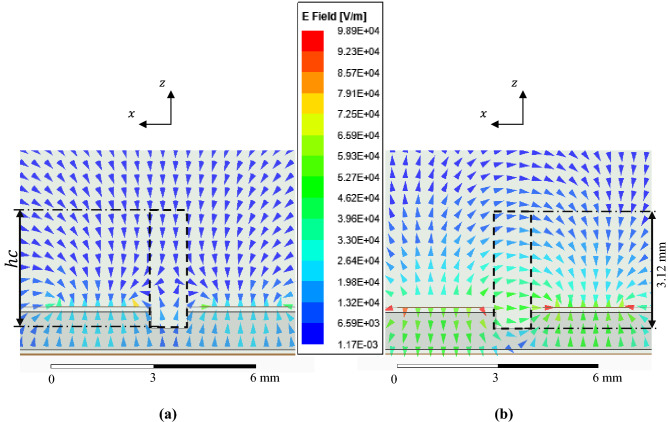
Electric field configuration at even (**a**) and odd (**b**) resonance frequencies, where it is shown that a SUT will be polarized vertically by the even mode and horizontally by the odd mode. Where the height of the cleft $hc$ was defined considering that the electric field is minimal at this distance and that the field lines maintain an adequate orientation for both modes.

The proposed sensor is shown in Fig. 1a, which consists of a pair of microstrip resonators coupled and separated by a distance $s$ known as the coupling region. The resonators are straight and open ended (i. e. half wavelength resonators). The sensor includes a cleft in the coupling region as shown in Fig. 1b. Where the electric field configuration is shown for the even mode (Fig. 2a) and for the odd mode (Fig. 2b) graphed in the cross-sectional view of the unloaded sensor (empty cleft) obtained by the Ansys full wave simulator (HFSS). Since the cleft is an open structure, it is important to define the height of the cavity $hc$ that will be considered in the present work. This was defined $hc$ = 3.12 mm consider that the electric field is minimal at this distance and that the field lines maintain an adequate orientation for both modes. The cleft can hold a SUT which will be exposed to electric field lines depending on the electric field configuration at the odd and even resonance frequency as shown in Fig. 2. A SUT placed in the cleft is polarized vertically (in the direction of the z-axis) when the even-mode resonance occurs (Fig. 2a), while it is horizontally polarized (in the direction of the x-axis) when the odd-mode resonance occurs (Fig. 2b). Therefore, by relating the real part of the permittivity with the resonant frequency and the imaginary part with the Q factor, it is possible to determine the complex uniaxial permittivity of a SUT.

### Methodology for the measurement of uniaxial permittivity using cavity perturbation theory

The resonant cavity disturbance method consists of introducing a small sample of material into a cavity with the intention of modifying the fields inside the cavity. For a dielectric SUT introduced in the cleft of the proposed sensor, the displacement in the resonance frequency with the ${\varepsilon {^{\prime}}}_{r}$ and the reduction of the Q factor with the $\varepsilon {^{\prime\prime}}$ can be related by Eqs. (3) and (4)

3 $$\frac{{f}_{1i}-{f}_{2i}}{{f}_{2i}}={A}_{i}({\varepsilon {^{\prime}}}_{rj}-1)\frac{{V}_{s}}{{V}_{c}}$$

4 $$\frac{1}{{Q}_{2i}}-\frac{1}{{Q}_{1i}}={B}_{i}{\varepsilon }_{j}^{{{\prime\prime}}}\frac{{V}_{s}}{{V}_{c}}$$

where $i=e, o$ indicates the odd or even mode, respectively, ${f}_{1i}$ and ${Q}_{1i}$, ${f}_{2i}$ and ${Q}_{2i}$ are the resonance frequency and Q factor before and after the disturbance, respectively. ${A}_{i}$ and ${B}_{i}$ are constant parameters independent of the dielectric characteristics of the sample. ${\varepsilon {^{\prime}}}_{rj}$ and ${\varepsilon }_{j}^{{{\prime\prime}}}$ ($j=\perp ,\parallel$) are the real and imaginary part of the relative permittivity of the SUT, ${V}_{s}$ is the volume of the SUT, while ${V}_{c}$ is the volume of the cleft. It is important to mention that Eqs. (3) and (4) have been taken from Chen et al.^16^ and adapted in our work for the electric field configurations at the odd and even resonance frequency of the proposed sensor.

Equations (3) and (4) are widely used for the measurement of complex permittivity for both isotropic and anisotropic samples. However, it is important to remember that such equations are obtained under the assumptions that: the resonator is empty at the initial condition (before perturbation), the disturbance in the resonator is made with a small sample compared to the size of the cavity, and the radiation losses are negligible. All of them are discussed and based on the perturbation theory made with small objects^17^. Among the main considerations that are made is that both the electromagnetic field configuration and the stored energy do not change with the introduction of the sample into the cleft. Under this statement, it is possible to consider the parameters Ai and Bi independent of the permittivity of the sample. The parameters Ai and Bi can be determined analytically, although this task is too complicated. Experimentally determining the parameters Ai and Bi can be carried out using Eqs. (3) and (4) in a calibration procedure measuring samples with known complex permittivity. But it should be noted that the known sample must be similar both in shape and location to the SUT.

**Figure 3:**
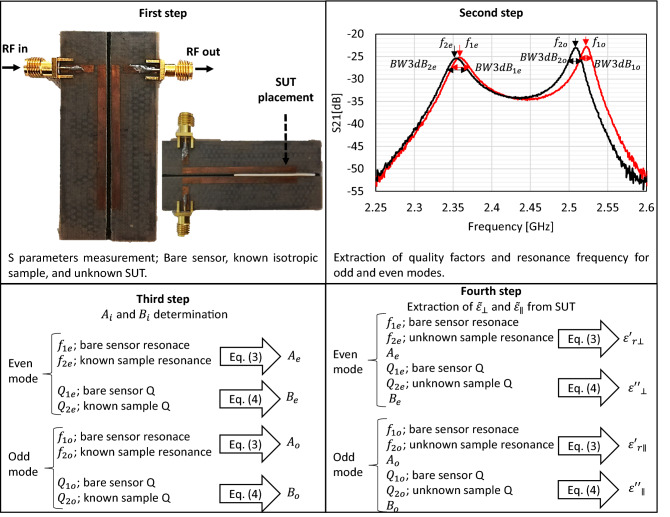
Uniaxial complex permittivity measurement methodology in flat solid samples using the cavity disturbance method for a microstrip sensor using coupled resonators.

The complex relative permittivity measurement methodology of a SUT is shown in Fig. 3 and is summarized in the following four steps:

**first step:** consists of measuring the sensor's S parameters for the following cases: (a) without sample, (b) with a known sample, and (c) with an unknown sample or SUT.

**second step**: consists of the extraction of the resonance frequencies and the Q factor for the even and odd modes. For this, the resonance frequencies are determined as the peak values of the transmission parameter S21 [dB]. The even mode occurs at the lowest frequency relative to the odd mode, which is the resonance that occurs at the highest frequency. The Q factor was determined using the -3 dB bandwidth for the two resonant modes.

**third step:** consists of determining the proportionality constants ${A}_{i}$ and ${B}_{i}$ using Eqs. (3) and (4), and the resonance frequencies and Q factors before the disturbance (case (a)), and after the disturbance with a known sample (case (b)).

**fourth step:** consists of determining the complex uniaxial permittivity of the SUT using the proportionality constants ${A}_{i}$ and ${B}_{i}$, the resonance frequency and Q factor of the unknown SUT (case (c)), and the resonance frequency and Q factor before the disturbance (case (a)).

It is worth noting that with the proposed methodology it is possible to obtain ${\stackrel{\sim }{\varepsilon }}_{\perp }$ through the even mode, and ${\stackrel{\sim }{\varepsilon }}_{\parallel }$ with the odd mode by measuring S parameters without the need for simulations or moving the SUT.

### Experimental measurement of uniaxial permittivity

The proposed sensor was implemented on a 3D printed substrate, using the PLA material with a deposit density of 50% through the honeycomb pattern. Thin films (0.127 mm dielectric thickness) of Cuclad 217 PCB material with ${\varepsilon {^{\prime}}}_{r}$=2.2 and $tan\delta$=0.0009 were used for the ground plane and sensor lines. With this, they were pressed against the PLA substrate to form the sensor. The volume of the cavity and of the sample are $vc=wc\times lc\times hc$ and $vs=ws\times ls\times hs$ respectively, where the width and length are delimited by the structure as shown in Fig. 1 (i.e., $ws\le wc$, and $ls\le lc$). As it is an open structure, it is important to clarify that the height of $hc$ is equal to 5 $D$ since at that height the field is minimal, the lines are well oriented and the disturbance is perceived simultaneously at the odd and even resonant frequency; additionally, the method requires $hs$ less than 3 $D$.

Measurements of the S parameters were performed with a VNA (Keysight FieldFox N9918A) under laboratory conditions at constant temperature. The sensor without sample has a measured even mode resonance of ${f}_{1e}$=2.3543 GHz ± 0.0008 and loaded quality factor ${Q}_{1e}$=63.372 ± 0.55. The odd mode resonance frequency was measured at ${f}_{1o}$=2.52425 GHz ± 0.0012 with a loaded quality factor of ${Q}_{1o}$=105.432 ± 0.89. The variations observed with the discharged sensor are mainly attributed to changes in temperature and relative humidity in the measurement environment.

In this work, we have used three different knowns materials to determine the proportionality constants ${A}_{i}$, and ${B}_{i}$ of Eqs. (3) and (4); the isotropic material PTFE, and the anisotropic ones; RT5870 and RO4003. Table 1 summarizes the resonance frequencies and quality factors measured with the loaded sensor consistently with each known sample. These values correspond to the average value with its standard deviation of twelve repeated measurements in each sample. It is important to mention that the three samples for the calibration were known using the method of Morales et al.^9^ for the real part of the permittivity tensor. While the imaginary part of the tensor was established equal to those reported in previous works^18–20^.

**Table 1:** Experimental resonant frequencies and quality factors obtained by the proposed sensor perturbed by different known samples.

Material	$ws$ (mm)	$hs$ (mm)	${f}_{2e}$ (GHz)	${Q}_{2e}$	${f}_{2o}$ (GHz)	${Q}_{2o}$	${\varepsilon {^{\prime}}}_{r\perp } {tan\delta }_{\perp }$	${\varepsilon {^{\prime}}}_{r\parallel } {tan\delta }_{\parallel }$	$\Delta {\varepsilon }_{A} \Delta {tan\delta }_{A}$(%)	Measurement frequency (GHz)
PTFE	0.96	1.54	2.3518	60.84	2.5045	103.58	2.051	2.051	0 0	2.43
			± 0.0012	± 0.606	± 0.0002	± 1.01	0.0012	0.0012		
RT5870	0.97	1.51	2.3462	61.36	2.5049	102.43	2.35	2.5	6.18	2.43
			± 0.0004	± 0.54	± 0.0003	± 1.05	0.0016	0.0023	35.89	
RO4003	1.03	0.79	2.3470	60.145	2.4951	98.291	3.37	3.67	8.52	2.43
			± 0	± 0.37	± 0	± 0.94	0.0029	0.0037	24.24	

The method was used to characterize anisotropic samples of printed PLA with 100% deposit density, Diclad 880, and RO4350B. Table 2 shows the dimensions of the SUT, resonance frequencies, quality factors, percentage of anisotropy and average measured value of the real and imaginary part of the permittivity tensor accompanied by its standard deviation calculated with the experiment repeated 12 times, at the temperature of 26 ± 0.27 °C, relative humidity of 53.95 ± 0.31, with the S parameters measured with the VNA Field Fox N9918A calibrated with the SOLT method using an IFBW of 100 Hz with a measurement step of 218 KHz , resulting in a frequency sweep time of 3 min per measurement. The printed PLA material was characterized using two different sample thickness with the proposed method and sensor by means of the known sample RT5870 defined in Table 1. For the PLA sample of $$hs $$ = 0.81 mm obtaining $${\varepsilon {^{\prime}}}_{r\perp } $$ = 2.70 ± 0.06, $${tan\delta }_{\perp }$$ = 0.0012 ± 0.0002, $${\varepsilon {^{\prime}}}_{r\parallel }$$ = 2.74 ± 0.01, $${tan\delta }_{\parallel }$$ = 0.002 ± 0.0007, $$\Delta {\varepsilon }_{A} $$ = 1.47%, and $$\Delta {tan\delta }_{A}$$ = 50% where the anisotropy percentages for the real and lost part are defined as $$\Delta {\varepsilon }_{A}=2({\varepsilon {^{\prime}}}_{r\parallel }-{\varepsilon {^{\prime}}}_{r\perp })/({\varepsilon {^{\prime}}}_{r\perp }+{\varepsilon {^{\prime}}}_{r\parallel })$$ and $$\Delta {tan\delta }_{A}=2({tan\delta }_{\parallel }-{tan\delta }_{\perp })/({tan\delta }_{\parallel }+{tan\delta }_{\perp }$$). Additionally, a second PLA sample with a sample thickness of 1.40 mm was characterized; the results obtained are $${\varepsilon {^{\prime}}}_{r\perp }$$ = 2.79 ± 0.06, $${tan\delta }_{\perp }$$ = 0.0017 ± 0.0002, $${\varepsilon {^{\prime}}}_{r\parallel }$$ = 2.69, $${tan\delta }_{\parallel } $$ = 0.0047 ± 0.0005, $$\Delta {\varepsilon }_{A}$$ = 3.65%, and $$\Delta {tan\delta }_{A} $$ = 93.75%. With this, we observe that for the two samples the results are close to each other for the two thicknesses studied and the real part of the uniaxial permittivity coincides with the work reported in Morales et al.^21^.

**Table 2:** Experimental results obtained by the proposed sensor and by previous works.

Material	$ws$ (mm)	$hs$ (mm)	${f}_{2e}$ (GHz)	${Q}_{2e}$	${f}_{2o}$ (GHz)	${Q}_{2o}$	${\varepsilon {^{\prime}}}_{r\perp } {tan\delta }_{\perp }$	${\varepsilon {^{\prime}}}_{r\parallel } {tan\delta }_{\parallel }$	$\Delta {\varepsilon }_{A}$ (%) $\Delta {tan\delta }_{A}$ (%)	Meas Freq (GHz)
PLA [this work]	0.95	0.81	2.3457	60.980	2.5002	101.730	2.70 ± 0.06 0.0012 ± 0.0002	2.74 ± 0.01 0.0020 ± 0.0007	1.47 50	2.43
PLA [this work]	0.85	1.40	2.3406	60.555	2.488	98.560	2.79 ± 0.06 0.0017 ± 0.0002	2.69 ± 0.02 0.0047 ± 0.0005	3.65 93.75	2.43
PLA^21^	25	3.42	N/A	N/A	N/A	N/A	2.73 –	2.79 –	2.17 –	2.4
PLA[22]	–	–	N/A	N/A	N/A	N/A		2.75 0.008	0	12
PLA^14^	–	–	N/A	N/A	N/A	N/A	2.75 0.0116	2.92 0.0118	6.00 1.71	40
Diclad 880 [this work]	0.92	1.51	2.3486	61.44	2.5037	102.62	2.13 ± 0.02 0.0011 ± 0.0002	2.28 ± 0.03 0.0022 ± 0.0004	6.80 66.66	2.43
Diclad 880^9^	10	3.18	N/A	N/A	N/A	N/A	2.17 –	2.33 –	7.11 –	2.4
Diclad 880^18^	–	–	N/A	N/A	N/A	N/A	2.15 0.0009	2.32 0.0016	7.6 53	12
RO4350B [this work]	0.96	0.75	2.3459	60.212	2.4960	99.508	3.55 ± 0.06 0.0030 ± 0.0005	3.73 ± 0.05 0.0034 ± 0.0003	11.47 131.03	2.43
RO4350B^9^	10	0.76	N/A	N/A	N/A	N/A	3.48 –	3.88 –	10.86	2.4

Subsequently, we had used the known istropic sample PTFE for characterize the material Diclad 880. We had ${\varepsilon {^{\prime}}}_{r\perp }$ = 2.13 ± 0.02, ${tan\delta }_{\perp }$ =0.0011 ± 0.0002, ${\varepsilon {^{\prime}}}_{r\parallel }$ = 2.28 ± 0.03, ${tan\delta }_{\parallel }$ = 0.0022 ± 0.0004, $\Delta {\varepsilon }_{A}$ =6.80%, and $\Delta {tan\delta }_{A}$ =66.66%. With these results, values for dielectric anisotropy close to those reported in previous works have been observed; at 2.4 GHz^9^ and 12 GHz^18^. 

Also, we have used the sensor to characterize RO4350B material by means the known RO4003 material. Obtaining: ${\varepsilon {^{\prime}}}_{r\perp }$ = 3.55 ± 0.06, ${tan\delta }_{\perp }$ = 0.003 ± 0.0005, ${\varepsilon {^{\prime}}}_{r\parallel }$ = 3.73 ± 0.05, ${tan\delta }_{\parallel }$ = 0.0034, $\Delta {\varepsilon }_{A}$=11.47%, and $\Delta {tan\delta }_{A}$ =131.03%. With these results, the dielectric anisotropy was measured close to reported by Morales et al^9^.

## Discussion

This work has presented a new sensor in microstrip technology that allows the characterization of the dielectric properties of materials with an anisotropic approach using a completely experimental measurement method. The sensor is based on a pair of coupled parallel line resonators and a cleft positioned in the coupling region to house the SUTs. This cleft allows to limit both the volume of the sample and the location of the sample in the sensor. Therefore, the field mode lines that penetrate the sample are purely horizontal when the odd mode occurs, and vertical with the even mode. This configuration allows the implementation of cavity perturbation techniques for the characterization of a uniaxial anisotropic SUT. Therefore, in contrast to Morales et al.^9^, in this new work the space-mapping algorithm is not required to determine the permittivity. The proposed methodology relates the change in the even/odd resonance frequency with the real part of the permittivity in the vertical/horizontal direction, and the change in the Q factor of the even/odd mode with the imaginary part of the permittivity in the vertical/horizontal direction. The characterization of different common anisotropic PCB dielectrics was carried out; Diclad 880, and RO4350B. As well as PLA 3D printed material.

Regarding the Diclad 880 material, the results reported here at 2.43 GHz are close to those measured in Morales et al.^9^ at 2.4 GHz where only the real part of the permittivity was measured obtaining ${\varepsilon {^{\prime}}}_{r\perp }$ = 2.17, ${\varepsilon {^{\prime}}}_{r\parallel }$ = 2.33, and $\Delta {\varepsilon }_{A}$ = 7.11%. On the other hand, in Dankov^18^ it was reported at 12 GHz that ${\varepsilon {^{\prime}}}_{r\perp }$=2.15, ${tan\delta }_{\perp }$ = 0.0009, ${\varepsilon {^{\prime}}}_{r\parallel }$ = 2.32, ${tan\delta }_{\parallel }$ = 0.0016, $\Delta {\varepsilon }_{A}$ = 7.6%, and $\Delta {tan\delta }_{A}$ = 56%. For the RO4350B material we can find at 2.4 GHz^9^ that ${\varepsilon {^{\prime}}}_{r\perp }$ = 3.48, ${\varepsilon {^{\prime}}}_{r\parallel }$ = 3.88, and $\Delta {\varepsilon }_{A}$ =10.86%. Where these small differences are attributed to local variations in the permittivity of commercial PCBs, in addition to the sample imperfections like non-nominal dimensions and the known material used to characterize the SUT’s. It is important to mention that since the present methodology is based on resonance disturbance techniques, to have a more accurate measurement, known samples with permittivity values as close as possible to the SUT are required.

For its part with the PLA material, it is important to mention that this thermoplastic is isotropic before being printed as measured in Dankov^22^ at 12 GHz. However, when this material is printed in 3D, anisotropy is induced in the printed material, even in the samples printed with a deposit density of 100% as measured in Morales et al.^21^ at 2.4 GHz, obtaining ${\varepsilon {^{\prime}}}_{r\perp }$ = 2.73, ${\varepsilon {^{\prime}}}_{r\parallel }$ = 2.79, and $\Delta {\varepsilon }_{A}$ = 2.17%. On the other hand, in Felicio et al.^14^ it was measured at 40 GHz that ${\varepsilon {^{\prime}}}_{r\perp }$ = 2.75, ${tan\delta }_{\perp }$ = 0.0116, ${\varepsilon {^{\prime}}}_{r\parallel }$ = 2.92, ${tan\delta }_{\parallel }$ = 0.0118, $\Delta {\varepsilon }_{A}$ = 6%, and $\Delta {tan\delta }_{A}$ = 1.71%. Note that the results are influenced by the resolution of the printer (manufacturing errors) and the frequency of measurement of the material. As the frequency of characterization increases, the wavelength begins to be comparable with the air grooves left between each deposited PLA filament^14^.

## Method

### Sensor fabrication and SUT

The sensor was implemented on a 3D printed substrate using PLA material and the Flashforge Finder 3D printer with precision in the z-axis direction of 2.5 µm and a precision in the x–y plane of 11 µm. The thickness of the printed substrate was 1. 316 mm using 50% deposit density by means of honeycomb mesh, with ${\varepsilon {^{\prime}}}_{r}$ = 2.47^21^. For the part of the ground plane and the power lines and resonators, the Cuclad 217 material with a thickness of 0.127 mm was used. So, the outer layers were pressed against the printed PLA to form the sensor shown in Fig. 3. Both the ground plane layer and the resonator layer were manufactured by photolithography.

The sensor consists of an input and an output feed network of width $w$ = 2.5 mm and length $lf$ = 10 mm. The transmission lines are separated from the resonators by a space $g$ = 1 mm. The resonators are open-ended with width $w$ and length $l$ = 45 mm separated by a coupling gap of $s$ = 1.2 mm. The dimensions of the cleft are $wc$ = 1.07 mm, $D$ = 0.62 mm, $lc$ = 65 mm and $hc$ = 3.12 mm. The height of the cavity $hc$ was defined as the height above the bottom of the slot to which the field lines are attenuated enough for a material to cause a disturbance in the sensor response (see Fig. 2). This cleft accepts solid samples of width $ws\le wc$ and height $hs$ preferably smaller than 3 $D$.

Diclad 880, RT 5870, RO4350B, RO4003 and PTFE samples were cut using the CNC laser cutting machine. For their part, the printed PLA samples were printed with 100% deposit density using the printer.

### The experiment

The entire measurement process was carried out under controlled laboratory conditions, at constant temperature (26 °C ± 0.27), pressure (101 kPa) and relative humidity (53.95 ± 0.31). For the measurement of the S parameters, the Keysight FieldFox N9918A vector network analyzer was used, performing a complete manual calibration of two ports (short, open, through, and match) before the measurements using an IFBW of 100 Hz and a resolution in frequency of 218 kHz. To avoid contamination of the samples the operator wore latex gloves during handling and subsequent placement of the samples on the sensor. Each sample was measured as shown in Fig. 3 in step 1, the sample was placed in the cleft from the middle of the coupled resonators to the opposite end of the feed lines. Using the transmission parameter S21 [dB], the resonance frequencies and Q factors for both modes were determined. The Q factor was determined by the bandwidth at 3 dB of the resonant frequency as follows: ${Q}_{\mathrm{1,2}i}=\sqrt{{f}_{l}{f}_{h}}/BW3dB$), where $BW3dB$ is the bandwidth at − 3 dB of the resonant frequency ${f}_{l}$ and ${f}_{h}$ are the frequencies lower and higher than − 3 dB. In this work it was considered that the measured loaded Q factors are equal to the discharged ones, due to the weak coupling with the transmission lines. The characterization results that were obtained are those shown in Table 2 where they are compared with other works found in the literature.
